# P04-04 Non-invasive biomarkers with high discriminant ability indicative of musculoskeletal health with ageing

**DOI:** 10.1093/eurpub/ckac095.058

**Published:** 2022-08-29

**Authors:** Sandra Agyapong-Badu, Martin Warner, Dinesh Samuel, Vasiliki Koutra, Maria Stokes

**Affiliations:** 1 School of Sport, Exercise and Rehabilitation Sciences, University of Birmingham, Birmingham, United Kingdom; 2 School of Health Sciences, University of Southampton, Southampton, United Kingdom; 3 Centre for Sport, Exercise and Osteoarthritis Research Versus Arthritis, University of Southampton, Southampton, United Kingdom

**Keywords:** Ageing, physical function, physical frailty, musculoskeletal health, screening

## Abstract

**Background:**

The use of large-scale pooled analyses and data sharing is a potential source to generate evidence to address complex scientific challenges and develop strategies to promote healthy ageing. However, the success of such analyses depends on robust measurements of musculoskeletal (MSK) health in ageing. Simple tests indicative of MSK health and suitable for use with older people are required. This study aims to assess the discriminatory ability of a combination of routine physical function tests and novel measures, notably muscle mechanical properties and ultrasound imaging of thigh composition (relative contribution of muscle and subcutaneous adipose tissue) to classify healthy individuals according to their age and gender.

**Methods:**

This cross-sectional study included 138 community-dwelling, self-reported healthy males and females (65 young, mean age±*SD* = 25.7±4.8 years; 73 older, 74.9±5.9 years). Handgrip strength; quadriceps strength; respiratory peak flow; timed up and go; stair climbing; anterior thigh tissue thickness (using ultrasound imaging), muscle mechanical properties (stiffness, tone and elasticity; Myoton technology); and self-reported health related quality of life (SF36) were assessed. Stepwise linear discriminant analysis was used to classify cases based on criterion variable derived from the known effects of age on physical function.

**Results:**

Combining conventional physical function tests with novel measures, revealed two discriminant functions which significantly (Wilks's λ = 0.05, 0.34; *p*>0.001) classified 89% of grouped cases with 11% error rate using leave-one-out cross-validation. Seven variables associated with grip strength, peak flow, timed up and go, anterior thigh thickness, and muscle mechanical properties demonstrated high discriminant ability (*p*>0.05 correlation with discriminant functions) to classify healthy people.

**Conclusions:**

The present study provides reference data for comparison with clinical populations and a comprehensive battery of non-invasive dry biomarkers with high discriminant ability indicative of musculoskeletal health. The most sensitive novel biomarkers require no volition, highlighting potentially useful tests for screening and monitoring effects of interventions on MSK health for vulnerable older people with pain or cognitive impairment. Older misclassified cases who appeared younger than predicted support the need for studies of older people with different habitual activity levels, to provide relevant reference values for assessment, so rehabilitation goals are targeted appropriately.

